# On Infanticide

**Published:** 1820-04

**Authors:** W. Hutchinson

**Affiliations:** Sackville-street


THE LONDON

### Medical and Physical Journal.

4 OF VOL. XLIII.] APRIL, 1820. [NO. 254.

"For many fortunate discoveries in medicine, and for the detection of numerous "errors, the world is indebted to the rapid circulation of Monthly Journals; "and there never existed any work to which the Faculty in EUROPE and "AMERICA were under deeper obligations than to the Medical and Physical "Journal of London, now forming a long, but an invaluable, series." RUSH.

### Original Communications, Select Observations, etc.

### MEDICAL JURISPRUDENCE.

### DISSERTATION II.—*On Infanticide.*

### § I. *On the Physiological Relations of Infanticide.*

[In continuation from page 187.]

THE changes of the colour and several other appearances of the lungs effected by means of respiration, were remarked by GALEN: he says, *Ob eam causam, substantia carnis pulmonis ex rubra, gravi, densa, in albam, levem, ac raram transfertur;** and the truth of these observations has been placed above disputation by succeeding physicians.† But this knowledge was not, it appears, applied to the objects of medical jurisprudence, until after this was inculcated by LANGIUS, RIVINUS, and SWAMMERDAM. At the time of AMMIANUS it seems not to have been effected; for he says, *Si submersio pulmonum est signum infallibile mortui fœtus protrusi ab utero, cur non secantur a medicis ordinarie, ut hac ratione infelices istæ puerperæ liberentur a tortura!*‡ BAGLIVI, who lived some years afterwards, was convinced of the security of the test above designated; his expressions on this point are absolute: *Pulmones fœtus mortui in utero matris, si extrahantur, et in aqua ponantur, petunt fundum; mortui vero extra uterum et aqua injecti innatant in ea. Quod signum ad infanticidia detegenda est evidentissimum.*§ MORGAGNI speaks of this experiment as of but recent adoption: *Haud scio tamen an cuipiam propterea in mentem venerit, ut ex hac re experimentum illud non nisi paucis ante meam ætatem lustris excogitaret.*||

* *De usu Partium,* lib. xv. c. 6.

† CHARLTON, in his disputation about the circulation of the blood, seems to have been the only physician who has denied the truth of the statement of Galen. See his *Exercit. Phys. Anat.* p. 207.

‡ *Praxis Vulner. lethal.* p. 410.

§ BAGLIVI, *op. omnia,* p. [illegible]99.

|| *De Sedibus et Causis Morborum,* Epist. xix. n. 45.

The principles of the application of the above-mentioned physical phenomena to the objects of medical jurisprudence, are the following: 1°, that full and free respiration, by means of which the texture of the lungs is more or less distended by air, effects such a change of the specific gravity of those organs in relation to that of water, as to cause them to float in this fluid; in which they sink when respiration has not taken place, unless certain other circumstances, which will be presently described, should have occurred; 2°, that *the signs of full and free respiration* are evidences of the existence of infantine life; 3°, that the non-establishment of respiration indicates, in a positive manner, the want of perfect development of infantine life.

These propositions point out the manner in which those phenomena are now regarded; but, the *floating* and *sinking of the lungs in water*, merely, were the things attended to when the experiment here indicated was first applied to medical jurisprudence, and the authority of those who inculcated it, led jurisconsults to acknowledge its validity, and to admit the results of it in evidence in forensic enquiries.* A critical examination of this measure, aided by pathological observations, at length placed its validity in doubt in the generality of cases, and proved its fallacy on several occasions. The observations of BOHNIUS,† HOFFMANN,‡ and HEISTER,§ demonstrated that the lungs of a fetus born dead will, under some circumstances, float in water, and those of one that has lived after its birth sink in the same fluid; and many other physicians have since had occasion to experience the correctness of those remarks. I shall treat, in succession, of all the important circumstances which relate to the validity of this *hydrostatic test*.

It has been shown in a former part of this dissertation, that it is possible for a fetus to respire whilst in the uterus, or when its head has been expelled without the vagina of the mother, though its body be retained; and that it may nevertheless have ceased to live at the instant of its birth, or total expulsion from the vagina. In these cases the lungs may float in water;‖ and this test will here furnish results similar to those obtained from the lungs of an *infant* that has respired.

It was observed, too, that the lungs might float in water in

---

* BEIERUS, *Deliniat. Juris. Crim.* MARCHIORI, *Miscellan. Criminal.*

† *De Offic. Med. De Vulner. renunciat.*

‡ *Op. Pathol. Pract.* tom. i.

§ *De Fallaci Pulmon. Infant. Experiment.*

‖ "If a child makes but one gasp, and instantly dies, the lungs will swim in water as readily as if it breathed longer, and had then been strangled. A child will very commonly breathe as soon as its mouth is born, or protruded from the mother, and in that case may lose its life before its body be born; especially when there happens to be a considerable interval of time between what we may call the birth of the child's head, and the protrusion of its body." HUNTER, *on the Uncertainty of the Signs of Murder in the Case of Bastard Children.*

consequence of putrefaction having taken place in them.* But it appeared from subsequent observations, that putrefaction must be very far advanced before this phenomenon will thence result; and before such a degree of putrefaction takes place in those organs, under ordinary circumstances, that of the body generally will have proceeded almost to the last stage. This was proved by CAMPER. "In order to ascertain," he says, "to what degree putrefaction would advance in an infant before its lungs would float in water, I made different experiments at Amsterdam on this subject; and I have found that, in those who had died before birth, the head might be so far decomposed by putrefaction, that the slightest force was sufficient to detach the bones of it from each other, as well as those of the arms and legs, before the lungs, which now began to participate in the putrefaction, would float in water."† These observations are derived from expositions of the bodies of infants in water as well as to the air on land: the results were similar. But, whilst the lungs remain free from this sort of decomposition, the state of the rest of the body need not prevent the enquirer from examining that of those organs, from which he may even now be able to derive proof of respiration having been effected; though this may be all the physiological evidence he can furnish to the court of judicature.

On making incisions into lungs in which putrefaction has made a certain degree of progress, we see air-bubbles forming strata between the ramifications of the bronchiæ, visible to the naked eye, which is never the case when the air-cells solely have been filled by respiration. But there is another mean of determining whether the air or gas diffused in the texture of the lungs has been introduced into them by respiration, or generated by putrid decomposition, that furnishes much more certain evidence. Portions of the lungs in this state should be firmly pressed between the fingers, or twisted in a folded cloth: if the air has arisen from putrefaction, the portions thus treated will sink in water, whilst portions of lungs that have been distended by respiration will float in it after very powerful pressure made in this way; that is, when the texture of the part has not suffered sensible decomposition from putrefaction. A lung dilated by respiration gives rise to a sort of crepitation on being compressed by the fingers or cut into by a scalpel, that is very

---

* ALBERTI (*De Pulmon. subsident.*) MORGAGNI (*loc. citat.*) HALLER (*op. Anat. Min.* t. i. p. 326. *Auctarium ad Physiol.* lib. viii. § iv. p. 37.) WRISBERG (*Nov. Comment. S. R. S. Gottingen,* tom. vi.) JAEGER (*Dissertat. de Fœtib. rec. natis.*) PLOUQUET (*loc. cit.* p. 272.)

† *Over de Oorzaaken van Kindermoord en van Zelfs moord.* Waar by twee *Proeven over de inblaazing der lucht in de longen van Kinderen welke dood ter waereld zyn gekomen.* Leeuwarden, 1744.

peculiar, and may be easily distinguished by an attentive observer who has once noticed it. This does not take place in the state of emphysema from putrefaction; nor will a certain degree of putrefaction prevent its being discerned in the lungs of an infant that has respired. Lecieux* states, that when the fetus has been extracted by the feet, especially when the pelvis has been very narrow, he has several times found a portion of the lungs float in water, although the fetus certainly had not respired, and died during the birth. He could not attribute this to putrefaction, because the body presented no signs of such a state, and he made his examination soon after its delivery. He conceives that the lungs had here suffered contusion, with extravasation of blood, from which bubbles of air had been disengaged; and hence the part immediately thus affected became specifically lighter than water. The accompanying circumstances already indicated must, in the greater number of cases, furnish the means for distinguishing this fact; and, should these fail, pressure of the part in question between the fingers will determine the question, by causing it to sink in water. The appearance of the air-bubbles will be also different, as was shown on a former occasion.

"It is so generally known that a child, born apparently dead," says Hunter, (who let nothing escape his penetrative views that was calculated to make the physician pause before he pronounced a criminative decision on this occasion,) "may be brought to life by inflating its lungs, that the mother herself, or some other person, might have tried the experiment. It might even have been done with a most diabolical intention of bringing about the condemnation of the mother." It has, however, been disputed whether or not this would cause the lungs to float, and the fact has even been denied by men of respectability. But no doubt should be entertained on this subject. Camper several times made the experiment of blowing air into the mouth of children, born dead, and who certainly had not respired, and he always found them readily expanded by this mean.† It succeeded also several times with Jager,‡ Schmitt,§ and Buttner. The last-mentioned physician had occasion to witness an instance in which this practice was really resorted to by a mother, and with the result just designated.‖ Augustin also relates a similar case.¶ But the other effects

* *Considerations sur l'Infanticide*, p. 55.

† *Eene grechtelyke en onleedkundige Verhandeling over de tekeneu van leven en dood in de nieuw gebooiene kinderen*, p. 86.

‡ Salzburg, *Medico-chirurgische Zeitung*, b. iii. p. 55.

§ *Neue Versuch und Erf über die Lungenprobe.*

‖ *Vom Kindermord*, p. 53.

¶ *Archiv. für Staatsarzneykunst*, i. p. 50.

of this measure are different from those produced by respiration. Insufflation of this kind, in a fetus that has not breathed, dilates the lungs, and renders their specific gravity less than that of water; but the arteries and veins are not dilated by this action, nor the absolute weight of these organs thereby augmented, which are the consequences of respiration. But here I anticipate a view of the subject, that should be taken after some other points have been discussed.

Another objection that has been made to the *hydrostatic test* of the lungs, is, that these organs will sink in water, in some cases, although the infant has lived and respired for several hours after its birth. Observations proving this fact have been made by HEISTER, MORGAGNI, HALLER, DE HAEN, HOFFMANN, ESCHEMBACH, KANNEGIESSERO, WRISBERG, PLENCK, PLOUQUET, LODER, BECKER, SCHOLL, KIEFER, MENDEL, PREU: and SCHENCK relates an instance* of an infant having lived four days, and cried several times, whose lungs sank to the bottom of a vessel of water. This circumstance may arise, either from some disease of the lungs of the tuberculous kind, or great sanguineous congestion, preventing more than a small portion of the lungs becoming dilated; or the same thing will ensue from want of sufficiently forcible respiratory efforts. Several of the physicians just named have well ascertained, indeed, that the lungs of infants are in general dilated gradually, and that many days often elapse before they are fully distended. The left lobe is very often not at all dilated for some days, as PORTAL and METZGER have proved. This fact had, indeed, been observed, long before, by BLANCARDI.† I have been informed by a late physician to the Foundling-hospital at Naples, who opened daily, on an average, the bodies of ten or twelve infants, which had generally died within twenty-four hours after birth, that he hardly ever found more than a very small portion of the lungs dilated by air: this portion was frequently not larger than a walnut in its green shell, and but rarely larger than a hen's egg, and it was commonly in the upper lobe of the right lung.‡ These facts show the necessity of making the hydrostatic test with portions only of the lungs, as well as with the organs in an entire state; because an infant may live with such a portion of the lungs dilated as will not render the specific gravity of the entire viscera less than that of water. I

---

* *Journal der Practischen Heilkunde,* Band. xxviii. seit. 3.

† *Anatom. Reform.* p. 71.

‡ It should be understood, that those children had never been fed before they were placed in the *turning-box* at the hospital; which, with the want of due warmth, &c. may have prevented their lungs being as much dilated as those of children of the same age are, perhaps, under ordinary circumstances.

have seen a case where the right lobe, when separated from the left, sank in water, though this was the most dilated by respiration, and the infant had lived forty hours, and cried strongly: but it died from suffocation by being *over-laid* (as it is popularly termed) by the mother, which had produced such an engorgement of blood in the organ, as to counterbalance the influence the small quantity of air it contained could have on its specific gravity. A piece somewhat more than a cubic inch in volume was the greatest portion that in this case floated.

But there are other circumstances of much greater importance pointed out by those facts: they show that there will often be much difficulty in determining whether or not a fetus has respired after its expulsion from the uterus; and that, when it has died within a short time after its birth, it may sometimes be impossible to decide this question from examination of the lungs alone; because, as already stated, it is proved that it may respire during the process of its expulsion from the uterus, and yet die before its complete delivery; and then the lungs may present appearances similar to those frequently witnessed after the lapse of several hours of *infantine* life.* Here the evidence collected from examination of other parts of the body will assist the medical enquirer in forming his opinions. If marks of destructive violence are any where present, it must be considered, not only whether they were calculated to destroy the life of the subject, but whether they must have been effected before or after its delivery from the mother, in order that he may determine whether *feticide* or *infanticide* has been committed. The situation of the effects of such violence, and the nature of it, will, in most cases, enable him to decide the latter question.

It appears, from the foregoing enquiry, that the only well-founded objection to the validity of the *hydrostatic test*, as a mean for the decision of the question, whether or not a *fetus* has *respired*, is that indicated by the possibility of *artificially* dilating its lungs with air, by blowing into its larynx;—a fact established by the cases cited in a former part of this dissertation. But this single objection would be sufficient to render the hydrostatic test devoid of utility in many instances, were there not known means for determining whether or not such a dilatation of the lungs was effected during life or after death; or,

* Some respectable writers have argued on the rarity of these cases, as a reason for neglecting them in forming our opinions on this subject: this is wrong. Had only a single instance of the fact alluded to been known to occur, it should be considered sufficient to prevent the physiologist regarding respiration as evidence of *infantine* life. It will, however, be proper for him to state the rarity of the fact to those whose duty it is to reason on the *moral* relations of the subject, in those cases of forensic enquiry where it might influence the juridical verdict.

more properly speaking, after the cessation of the action of the heart and the circulation of the blood.

More precise and comprehensive views of the changes effected in the lungs by respiration, at length showed that the alteration of the specific gravity of those organs in relation to their volume was not the only mean they furnished for determining the question under consideration. The establishment of respiration is accompanied with an increase of the flow of blood to the lungs: the pulmonary arteries, which before this was effected had been in a collapsed state, now become dilated; their ramifications in the lungs become full of blood, and a considerable addition to the *real weight* of the lungs is thus made. These changes cannot be produced by artificial insufflation after death, or after the action of the heart and the circulation of the blood have ceased. Besides this dilatation of the pulmonary arteries, the colour of the lungs is much altered: from having been of a deep, dullish red, those organs assume a paler, but more vivid, red hue; and the blood in the vessels is commonly frothy. With these appearances may be witnessed, if respiration has been established for a certain length of time, a collapse, and more or less complete destruction, of the *canalis arteriosus;* perhaps closure of the foramen ovale in the septum of the auricles of the heart. The state of the emphysema of the lungs, and their development so as to spread over and nearly cover the pericardium, are important circumstances, only in connexion with the preceding ones, in our present view of this subject, because the same effects may result from artificial insufflation. The latter remark is equally applicable to the vaulted appearance of the anterior parietes of the thorax, and the lessening of the concavity of the figure of the diaphragm; though, when this muscle has been merely pressed lower into the abdominal region by dilatation of the lungs after death, it may be made to return to its original state of concavity by a little pressure after the lungs are removed from the thorax, which cannot be done after it has properly *contracted* by its vital efforts. Therefore, by carefully noticing the level, with respect to the ribs, of the central tendon of this muscle, and making the experiment just designated, some useful evidence may often be acquired: though more precise knowledge than we yet possess, respecting the situation of the summit of the diaphragm before and after certain stages of respiration, is requisite, in order to render this evidence of much utility. I shall now show in what manner the facts just narrated have been applied to the object of jurisprudence under consideration.

The lungs of the fetus are small, compact, and contain but little blood, the pulmonary, arteries being nearly collapsed; but, as already stated, when respiration is established, the pulmonary arteries become dilated, an increased afflux of blood takes place to these organs, and their *real* weight, as well as their volume, is considerably increased. It is on these principles that Plouquet* founded his mean for procuring additional evidence respecting the existence of respiration. He found, on examination, that the body of a male infant born dead, and which had not respired, weighed 53040 grains, comprising the lungs, and that these organs alone weighed 792 grains: the proportion of the lungs to the body was, then, as 1 to 67. In another infant, he found the proportion as 1 to 70. He then examined a third, born at the full period, and which had respired: here the proportion of the weight of the lungs to that of the body, was as 2 to 70.

Some objections of but little import were first advanced against this *assay* (it is thus termed) of Plouquet. Thus Metzger† remarked, that Plouquet had paid no regard to the effects of hemorrhage from the navel-string; but Jager replied to this,‡ by saying that hemorrhage would affect the whole system in the same ratio, or nearly so, as the lungs, and then the same relative weight between them would still exist. But a greater difficulty was advanced by Haartmann,§ who stated that he had not found the relation of the weight of the body to that of the lungs even nearly similar to that mentioned by Plouquet; and the latter confessed that he had founded his statement on the examination of only three bodies. Haartmann gives about 48 to 1 as the proportion after respiration has been effected, and about 59 to 1, as that existing before respiration. Struve stated, that he had found no constant relation between the weight of the lungs and the body under these circumstances;|| and the experiments of Schmitt showed similar results. The reason of this diversity, without considering the influence of variation in the original construction of the body, is sufficiently accounted for by the great diversity in the manner in which respiration is established in newly-born infants. It seems to be proved that, in a great proportion of infants, it is effected but gradually and slowly, and that several days elapse before the lungs are fully dilated. Nevertheless, the *assay* of Plouquet must be considered of some utility, especially in addition to the evidence deduced from the state of the pulmonary arteries, with the other phenomena resulting from respiration; but, before it can be of much importance, it will be necessary that a

* *Commentarius in Processus Criminales,* sect. ii.

† In *Loder's Journal,* ii. 141.

‡ *De Vita Neogeni,* p. 38.

§ Stockholm, *Acad. Handl.* tom. xx. p. 40.

|| *Dissert. de Docimas. Pulm. Ploucq.*

maximum and minimum of the relative weight of the body and the lungs, should be established from sufficiently-extensive series of experimental observations.* LECIEUX relates the results of four hundred examinations of bodies of children, made at the Hospice de la Maternité at Paris, for the purpose of furnishing some evidence on this subject; and the results of them are almost as various as it was possible for them to have been, within a certain range. This list furnishes but few instances of fetuses born dead at the full period without some unusual appearances of the lungs, (as putrefaction, &c.) and, therefore, but little evidence on one very interesting point. It however appears from it, that, in one instance, the proportion of the weight of the body to that of the lungs, was no greater than 27 to 1, (that of the body being 1958 *grammes*, which is below the medium,† this being about 2400,) in a male infant of the full term, born dead, and presenting no remarkable appearances; and there were several cases of fetuses, born under the same circumstances, except that the weight of the body was of the medium standard, presenting nearly as low a ratio. The proportion was as low as 10 to 1 in one instance, in a male infant of the full term, weighing 2600 grammes, which lived eleven days. No remarkable appearances were noticed in the body. In a male fetus of eight months, born dead, presenting no remarkable appearances, the proportion was as great as 131 to 1; the weight of the body being 1836 grammes. In several instances, under similar circumstances, in females as well as in males, the proportion was from 80 to 100 to 1. In a female of the full term, dead born, presenting no remarkable appearances, weighing 2570 grammes, the ratio was 86 to 1. In another, that died during labour, which was very long, the proportion was 94 to 1; the weight of the body being 3000. In a male, dead-born at the full term, with nothing remarkable, the weight of the body being 3672, the proportion was as high as 90 to 1. In a male infant of the full term, who lived four days, weighed only 2150 grammes, and where no remarkable appearances were noticed in the body, the proportion was as 48 to 1. In another instance it was 43 to 1, at the age of ten days, where the weight of the body was 2250 grammes. But, under similar circumstances, it rarely exceeded 40, even after only a few hours of infantine life; and it generally was about 30 or 35, in those who lived from three or four to eight or ten days, the body having about the medium weight. These observations are sufficient to show how difficult, and indeed apparently impossible, it will be to establish any standard in regard to the *assay*

* M. MARC states, that he and M. GILBERT are engaged in the enquiry here indicated.

† One gramme is equivalent to 18°·837′ grains.

of Plouquet, that can be applied to it with precision. I need only point out, that the minimum of the ratio of the weight of the body to that of the lungs before respiration, often passes below the common ratio after respiration has been established, and occasionally extends nearly as low as the minimum of the latter; so that the instances in which any standard could be relied on in an absolute manner must here be extremely rare. On the other hand, the ratio after respiration has been established, is often higher than the medium ratio before respiration; and therefore the same difficulties present themselves in this as in the former view of the subject.

It appears, then, from the foregoing examination of the *assay* of Plouquet, that it will, generally, furnish useful evidence only in connexion with other circumstances: that is, when emphysema of the lungs apparently arising from *respiration*, and the other phenomena already mentioned as consequences of the establishment of that function exist, then the results of this *assay* may furnish such evidence as will tend to confirm what those phenomena indicate.

It is necessary to speak here of the tests proposed by Daniel, though one of them is founded on the same principle as that of Plouquet, and the other cannot be relied on with much confidence. He stated, that we might judge of the existence of respiration by the increase of weight given to a certain quantity of water in which the lungs had been strongly compressed, as this would gain what the lungs lost by the expulsion of the fluid they contained. All the objections to the measure of Plouquet apply still more forcibly to this of Daniel. The other test is founded on the increased circumference of the thorax, and the vaulted appearance it assumes after respiration has been established. The circumference of the thorax varies so much in infants of the same age and sex, both absolutely and in proportion to other parts of their body, that it cannot be possible to obtain any decisive evidence from this mean. The vaulted appearance of the chest is almost equally fallacious in the generality of cases, or else it is devoid of utility; because the figure of the thoracic parietes is not much changed until respiration has been fully established, and then we have other and more certain means of detecting its existence. Besides this, it appears from the experiments of Schmitt,* that the thoracic parietes were distended outwards by artificial insufflation after death, as much as they are by actual respiration as it occurs in the newly-born infant: and yet some respectable men have been so rash as to assert, that they could determine, by inspec-

* *Neue Versuch und Erf über die Lungenprobe.*

tion of the exterior of the chest alone, whether or not an infant had respired.

Having thus given the theory of the different tests to which the lungs may be submitted, in relation to the subject of forensic enquiry, I shall resume the didactic manner of the first part of this dissertation, and return to the place where the medical practitioner was about to open the chest of the body before him, that I may show how the knowledge above developed should be practically applied.

The body should now be weighed, if means for effecting this can have been procured. The state of the thoracic parietes should then be observed, in regard to their extent and figure; especially whether they are flat, or vaulted anteriorly.

The cavity of the chest is then to be exposed in the usual manner,* and with the utmost care not to cut or destroy unnecessarily any of the parts it contains. The situation of the different organs exposed to view should then be accurately noticed; especially whether the lungs are collapsed or dilated, and whether they cover the lateral parts of the pericardium, and have filled the vacancy in the thorax left by the other organs. Any signs of disease that may be present are to be carefully examined; and if blood, or purulent or other analogous matter, be effused in the cavity, its origin should be sought for, and the cause of its presence determined as exactly as possible. If any blood-vessels, or the heart, are found ruptured, care must be taken to ascertain whether this can be traced to external violence, and whether this arose from wilful and criminal means, or the act of parturition. Ligatures may now be put on the aorta and venæ cavæ, near their attachment to the heart; the trachea divided close to the bronchiæ; the vessels cut beyond the ligatures; and the heart and lungs, attached together, then removed from the cavity of the thorax. If bloody, they should be cleansed with a sponge; and then the colour of the lungs, their consistence and elasticity, and their state with regard to healthy structure, distinctly noticed, without compressing them forcibly, or lacerating in any way their structure. If the body generally is in a state of putrid decomposition, it should be ascertained whether the lungs are also thus affected, and in what degree. The lungs are to be turned with the bronchial trunks downwards, that any fluid which may be contained in these tubes may flow out, and then weighed, in conjunction with the heart.

* The method of dissecting dead bodies, in relation to forensic enquiries, will be considered in a particular manner in the dissertation on *Homicide*.

A vessel, of a foot or a little more in diameter, and of at least a foot and a half in depth, is to be filled to the height of not less than a foot with pure, *fresh*, and, if possible, *river* water, the temperature of which should be nearly equal to that of the air, unless this be very cold or very hot. The lungs and heart, still attached together, are to be placed in a gentle manner in this water. It must then be remarked, whether they float near the surface of the water, or sink to the bottom; whether they fall suddenly, or descend slowly; whether the lungs turn uppermost, and float near the surface of the water, or about the middle of the fluid.

The heart is now to be separated from the lungs, having previously applied a ligature to the pulmonary vessels, to prevent the escape of the blood they may contain; and the weight of the heart alone then determined, that it may be subtracted from that of the heart and lungs together, previously ascertained.

The lungs are now to be placed alone in the water; and great attention must be paid to the position they assume in it: that is, whether they rapidly or slowly sink, or float near the surface; whether, by reversing their vertical situation in the water, they sink more readily or with more difficulty; and, if any part constantly rises and is drawn under water by the rest, this part should be particularly marked.

The two lobes must be separated, and the above-mentioned experiment made with each distinctly, and any difference in the results remarked: if one lobe float and the other sink, it should be noticed whether it is the right or left that floats. Each lobe is then to be cut into several pieces, taking care not to confuse those of the right with those of the left; and the following circumstances attended to whilst this division is effected: Whether or not there is the crepitation which is always heard on pressing a lung dilated by respiration; but it must be borne in mind, that a similar crepitation may arise from a lung artificially dilated by air. If emphysema be present, whether the air exists in the proper air-cells, or is diffused in the general texture of the lungs; and if this texture be dilated into visible globules by the air. Whether the vessels contain much or little blood, or if they, especially the branches of the proper pulmonary arteries and veins, are entirely devoid of it: if they are, whether the other blood-vessels are also empty, because this state of vacuity of the lungs may exist as a consequence of hemorrhage, though respiration has been established. Portions of them should be pressed under water, in order to discover whether bubbles of air escape from them. The state of their structure, with regard to health and disease, may be again examined. Each portion should be separately placed in water; and these portions are to be put in, first of a moderate volume, and then after having been cut into small pieces; and, lastly, after having suffered forcible compression between the fingers. If putrid decomposition has taken place in them, portions of other organs of a somewhat similar structure, as the spleen and the liver, should also undergo the hydrostatic test.

The large blood-vessels must now be carefully examined, especially the veins and the canalis arteriosus, and lastly, the heart itself, to ascertain if it presents any morbid appearance, and if the foramen ovale of the auricles remains fully open.*

*Sackville-street; March 4th,* 1820. W. HUTCHINSON.

* This dissertation is again interrupted, from the necessity for confining this subject to a certain portion of the limits of the Journal; but it will be completed in the next Number.

